# Supplementary material for: Modeling glioblastoma heterogeneity as a dynamic network of cell states
Source: Mol Syst Biol. 2021 Sep 16;17(9):e10105. doi: 10.15252/msb.202010105 (PMC8444284; doi:10.15252/msb.202010105)
Supplement: Supplementary file 6 — Source Data for Figure 5 [file MSB-17-e10105-s004.zip › Figure5A_sourcedata/GSEA_3017/nosigdb_stateB.GseaPreranked.1622795021725/RICHARDS_VERHAAK_CANCERCELL_2010_PRONEURAL.html]

Details for gene set RICHARDS\_VERHAAK\_CANCERCELL\_2010\_PRONEURAL[GSEA]

|  || Dataset | state43017 |
| Phenotype | NoPhenotypeAvailable |
| Upregulated in class | na\_neg |
| GeneSet | RICHARDS\_VERHAAK\_CANCERCELL\_2010\_PRONEURAL |
| Enrichment Score (ES) | -0.4133032 |
| Normalized Enrichment Score (NES) | -2.1784117 |
| Nominal p-value | 0.0 |
| FDR q-value | 0.0058306735 |
| FWER p-Value | 0.014 |
Table: GSEA Results Summary

  

Fig 1: Enrichment plot: RICHARDS\_VERHAAK\_CANCERCELL\_2010\_PRONEURAL      
 Profile of the Running ES Score & Positions of GeneSet Members on the Rank Ordered List

  

| PROBE | GENE SYMBOL | GENE\_TITLE | RANK IN GENE LIST | RANK METRIC SCORE | RUNNING ES | CORE ENRICHMENT || 1 | RAD21 |  |  | 115 | 0.506 | -0.0757 | No |
| 2 | ATAD5 |  |  | 170 | 0.442 | -0.0794 | No |
| 3 | NCAM1 |  |  | 178 | 0.436 | -0.0207 | No |
| 4 | DPF1 |  |  | 264 | 0.367 | -0.0777 | No |
| 5 | TOPBP1 |  |  | 369 | 0.319 | -0.1679 | No |
| 6 | CDKN1B |  |  | 427 | 0.298 | -0.1981 | No |
| 7 | TTYH1 |  |  | 469 | 0.287 | -0.2084 | No |
| 8 | ARHGAP33 |  |  | 482 | 0.285 | -0.1800 | No |
| 9 | ADGRL3 |  |  | 537 | 0.274 | -0.2098 | No |
| 10 | DCX |  |  | 689 | -0.264 | -0.3720 | Yes |
| 11 | CHD7 |  |  | 706 | -0.284 | -0.3490 | Yes |
| 12 | MMP16 |  |  | 707 | -0.285 | -0.3045 | Yes |
| 13 | MYO10 |  |  | 710 | -0.305 | -0.2594 | Yes |
| 14 | SOX4 |  |  | 712 | -0.305 | -0.2130 | Yes |
| 15 | MARCKSL1 |  |  | 716 | -0.311 | -0.1683 | Yes |
| 16 | DLL3 |  |  | 736 | -0.414 | -0.1291 | Yes |
| 17 | MLLT11 |  |  | 744 | -0.430 | -0.0712 | Yes |
| 18 | SOX11 |  |  | 754 | -0.576 | 0.0067 | Yes |
Table: GSEA details [plain text format]

  

Fig 2: RICHARDS\_VERHAAK\_CANCERCELL\_2010\_PRONEURAL: Random ES distribution      
 Gene set null distribution of ES for **RICHARDS\_VERHAAK\_CANCERCELL\_2010\_PRONEURAL**
